# Determination of the Ionic Association Constants of Na^+^ with CO_3_^2−^ and HCO_3_^−^ Ions, in NaCl-NaHCO_3_-H_2_O Ternary Systems, at 25 °C

**DOI:** 10.3390/molecules28196813

**Published:** 2023-09-26

**Authors:** Mihaela Ganciarov, Rusandica Stoica, Ana Maria Josceanu

**Affiliations:** 1 Analysis Department, National Institute of Chemistry and Petrochemistry R&D of Bucharest, 202 Splaiul Indepentei Street, 060021 Bucharest, Romania; mihaela.ganciarov@icechim.ro (M.G.); rusandica.stoica@icechim.ro (R.S.); 2 Department of Analytical Chemistry and Environmental Engineering, National University of Sciences and Technology Politehnica Bucharest, 1-5 Polizu Street, Sector 1, 011061 Bucharest, Romania

**Keywords:** activity coefficients, ternary system, association constant, effective ionic strength, algae growth media

## Abstract

The ionic association constants of sodium with carbonate ion ($K_{1C}^{\prime }$) and acidic carbonate ions ($K_{2C}^{\prime }$) were measured in NaCl-NaHCO_3_-H_2_O ternary systems to determine the distribution of sodium among the chemical species present in the growth medium of *Chlorella homosphaera 424* algae. The mean activity coefficients of sodium chloride (in pure sodium chloride and in a mixture of electrolytes) were determined experimentally using two electrochemical cells, namely Ag, AgCl| KCl (3 M)|| NH_4_NO_3_ (1 M)| NaCl ($m_{\mathrm{NaCl}}$)| Na^+^-ISE and Ag, AgCl|KCl (3 M)|| NH_4_NO_3_ (1 M)| NaCl ($m_{\mathrm{NaCl}}$)| Cl^−^-ISE. The studies carried out show that the values of the association constants of $K_{1C}^{\prime }$ and $K_{2C}^{\prime }$ do not depend on the composition of the medium, but only on the effective ionic strength. The experimentally obtained $\gamma_{\mathrm{NaCl}}^{0}$ values in the binary system are comparable to the mean activity coefficients values for NaCl, calculated using data from the literature, with −0.9 to 0.1% relative standard deviation. The obtained results show that the experimentally determined mean activity coefficient in the ternary system, $\gamma_{\mathrm{NaCl}},$ is smaller than $\gamma_{\mathrm{NaCl}}^{0}$ in the binary system over the entire field of ionic strengths studied. The ternary system NaCl-NaHCO_3_-H_2_O obeys Harned’s rule.

## 1. Introduction

The studies carried out and presented in this paper are part of the general topic of interest of reducing the greenhouse effect that is generated globally by emission of gases such as carbon dioxide, methane, nitrous oxide, chlorofluorocarbons (CFCs), and sulfur dioxide. Currently, the most important greenhouse gas is carbon dioxide [1,2].

The accumulation of carbon dioxide in the atmosphere, with all its negative consequences for human society, is both a reality and a challenge for science and technology today. The warming of the atmosphere is due to carbon dioxide emissions in a proportion of 52%. The main cause for the increase in the level of carbon dioxide in the atmosphere is the use of fossil fuels for energy generation, transport, industry, and domestic utilities.

To reduce carbon dioxide emissions to the atmosphere, many technological solutions have been advanced, including geological sequestration, oceanic sequestration, biological sequestration, and mineral sequestration [1,3,4,5,6,7]. A relatively recent biological solution for reducing the harmful effects of carbon dioxide emissions in the atmosphere is its sequestration in the form of biomass, using microalgae. Microalgae are present in the entire ecosystem, not only in the aquatic environment, but also on land, presenting great variety. It is estimated that there are more than 50,000 species, but only around 30,000 have been studied and analyzed [8,9].

Microalgae have an interesting and completely unexploited potential in biotechnology. They synthesize products such as natural dyes, polyunsaturated fatty acids, polysaccharides, and vitamins. As one of the largest groups of oxygen producers worldwide, microalgae play an important role in ecosystem balance [3]. After extraction, the spent material can be used as fertilizer, fish food, etc.

Biologically, the selection of microalgae species that can be used for the efficient sequestration of carbon dioxide from industrial emissions is not at all simple, because they are different both in morphology, and during the growth period [10].

The cultivation of microalgae requires real-time analytical control of the chemical species present in the specific culture media, which are complex synthetic solutions of electrolytes with a composition that is significantly different to that of natural waters/media, especially due to the high concentration of acidic carbonate ions; these can vary in the 0.10–0.50 mol/L range, in the case of a 0.2–0.55 mol/L total ionic strength [10], when the ionic species are closely associated.

The nutrient growth medium for *Chlorella homosphaera 424* is medium Z (Zarrouk) [10], consisting of high levels of NaHCO_3_, accompanied by K_2_HPO_4_, NaNO_3_, K_2_SO_4_, NaCl, MgSO_4_×7H_2_O, and CaCl_2_×2H_2_O. In situ monitoring of the species distribution is a critical step for avoiding inactivation of microorganisms and offering optimal development. Potentiometric measurements might deliver real-time information that is useful for building a species distribution diagram, provided that all possible interactions and associations are taken into account. Thermodynamic constants describing such processes have been reported in the literature, generally at ionic strengths higher than 0.5 mol/L. Developing a procedure for anionic and cationic species determination, by direct or indirect potentiometric measurements, in the culture media of *Chlorella homosphaera 424* algae could start with studying the media influence on the values of the association constants for ternary mixtures of the compounds present (NaCl-NaHCO_3_-H_2_O NaCl-Na_2_SO_4_-H_2_O, and NaCl-Na_2_HPO_4_-H_2_O), before approaching the multicomponent system (NaCl-NaHCO_3_-Na_2_HPO_4_-Na_2_SO_4_-H_2_O).

Software packages have often been used for chemical speciation calculation and graphical representation in the literature (HySS 2009 [11,12], Aq_solution 3.2 [13], KEV 3.0 [14]). Generally, input data for the specialized applications are the reactants’ total concentrations, the association constants, and the solubility products.

There are no values reported in the literature for the association constants in the NaCl-NaHCO_3_-H_2_O ternary system at ionic strengths lower than 0.5 mol/L. Kester and Pytkowicz [15,16] used a method based on electromotive voltage measurement to determine the association constants in sodium chloride- and sodium sulfate-containing solutions at hydrogen ion concentrations where the HSO_4_^−^ type ionic species are negligible, using a 0.638–0.690 mol/L effective ionic strength. The activity of the free cation (Na^+^) was measured in standard sodium chloride solutions and solutions containing NaCl-Na_2_SO_4_. The authors demonstrated that the association constants do not depend on the composition of the medium in which they were determined, but on the effective ionic strength of the medium. The reproducibility of their results decreases with increasing sodium chloride concentration.

Butler and Huston [17,18,19] determined the ionic association constants of NaHCO_3_ and NaCO_3_^−^ in the presence of sodium chloride in cells without liquid junction, in the 0.5–3 mol/L ionic strength range.

Pytkowicz and Hawley [20] measured the apparent association constants for the formation of acidic carbonate and carbonate ion pairs with Na^+^, Ca^2+^, and Mg^2+^ at 25 °C and 0.72 mol/L ionic strength. The results were used, together with the sulfate ion association constants obtained by Kester and Pytkowicz [16], to determine the major ionic species in seawater at 25 °C and 34.8% salinity. Capewell, Hefter, and May [21] determined the association constant of NaCO_3_^−^ in CsCl medium in the 0.5–7.0 mol/L ionic strength range and at 25 °C, using sodium ion-selective electrodes. Blum [22] proposed a new method for determining ion association constants in KCl-Me_4_NCl and Me_4_NCl media, using ion-selective electrodes at various ionic strengths (Table 1). Chung Yan-Chun [23] determined the stoichiometric association constant of NaCO_3_^−^ in the 10–40 °C temperature range and the 0.20–0.62 mol/L ionic strength range in NaCl-[(CH_3_)_4_N]_2_BPDS-Me_4_NCl medium, and the stoichiometric association constant of NaHCO_3_ in the 10–40 °C temperature range and 0.20–0.62 mol/L ionic strength range in NaCl-Me_4_NCl medium.

A synthesis of the experimental values of the association constants of sodium with carbonate and carbonate acid ions available in the literature is presented in Table 1.

The purpose of this work is to evaluate the ionic association constants of sodium ion with carbonate and acidic carbonate ions in ternary systems of the NaCl-NaHCO_3_-H_2_O type at 0.1–0.5 mol/kg total ionic strength, to determine the distribution of sodium among the chemical species present in the growth medium of *Chlorella homosphaera 424* algae, also known as “sodium speciation”. This paper is part of a larger study which has as main objective to develop a procedure for the determination of species (anionic and cationic) by direct or indirect potentiometric measurements, in situ or on samples taken from the culture media of *Chlorella homosphaera 424* algae.

Sodium speciation in the NaCl-NaHCO_3_-H_2_O ternary system has been approached through the ionic association model as modified by Pytkowicz and Kester [16]. It is based on the hypothesis that the change in the mean activity coefficients of the salts present occurs only as a result of the formation of ion pairs. The verification of this hypothesis has been carried out by preliminarily studying the behavior of the NaCl-NaHCO_3_-H_2_O ternary systems in relation to Harned’s rule.

## 2. Results and Discussion

The analytical control of a biological carbon dioxide sequestration process involves two stages. The first stage of the carbon dioxide sequestration process is its capture by absorption in an aqueous solution of sodium carbonate. The chemical species present in the absorption medium are CO_3_^2−^, HCO_3_^−^ NaCO_3_^−^, NaHCO_3_, and H_2_CO_3_, and physically dissolved CO_2_, Na^+^, H_3_O^+^, and OH^−^. The control of the absorption process involves the monitoring of the chemical species in the solution, and sometimes in the gas phase. The experimental data obtained in the first stage were the subject of a previous study [24].

The second stage of the carbon dioxide sequestration process is storage of CO_2_ in the form of microalgal biomass. In this step, the acidic carbonate solution obtained in the first step is diluted and fertilized to create a suitable culture medium for the growth of *Chlorella homosphaera 424 algae*. A typical *Chorellahomosphaera 424* growth media composition [10] consisted of 16.80 g/L NaHCO_3_, 0.5 g/L K_2_HPO_4_, 2.5 g/L NaNO_3_, 1 g/L K_2_SO_4_, 1 g/L NaCl, 0.2 g/L MgSO_4_×7H_2_O, 0.04 g/L CaCl_2_×2H_2_O, 0.05 g/L chelated iron, and microelements in a concentration below 0.003 g/L. The microelements mix contained H_3_BO_3_, MnSO_4_·4H_2_O, ZnSO_4_×7H_2_O, MoO_3_, CuSO_4_×5H_2_O, and Co(NO_3_)_2_×6H_2_O.

The association constants of sodium ions with the anionic species present in the algal media growth were determined simultaneously with the concentrations of all species, by solving the mathematical model of the equilibrium state for the electrolytic system. The equilibrium state of the electrolyte solution is described by a system of equations including mass balance equations, neutrality conditions (electric charge balance), and chemical equilibrium relations for the present species. 

The model is based on three simplifying hypotheses: (*i*) the anionic species with which sodium ions can associate in the solution are CO_3_^2−^, HCO_3_^−^, H_2_PO_4_^−^, HPO_4_^2−^, and SO_4_^2−^ and the influence of the anions present in micro-concentrations is negligible; (*ii*) the salts of strong acids such as sodium chloride, sodium nitrate, and sodium sulfate are considered completely dissociated; and (*iii*) mean activity coefficients of the salts change only as a result of the formation of ion pairs, provided that the total ionic strength is kept constant.

Standard sodium solutions, also used by J. N. Butler [18] and R. M. Pytkowicz [16], were considered as reference systems, because they present a number of advantages. The associations between sodium and chloride ions are negligible and there are commercial and reliable ion-selective electrodes, both for sodium and chloride. In addition, sodium chloride is an intensively studied electrolyte, so there is information in the literature on its mean activity coefficients, obtained by various procedures, with appropriate uncertainties.

In order to evaluate the magnitude of the associations between sodium ion and the CO_3_^2−^ and HCO_3_^−^ species, the influence of the composition on the activity coefficients of sodium chloride was studied at constant ionic strengths, and Harned’s coefficients (α_12_) [25] were determined in NaCl-NaHCO_3_-H_2_O ternary electrolyte systems. Both the mean activity coefficients, obtained experimentally, and database [26] values were used.

The association constants were determined as functions of the effective ionic strength in NaCl-NaHCO_3_-H_2_O ternary systems. In the pH range close to neutral, in which algae growth occurs, carbonic acid is completely dissociated and presents two differently protonated forms, CO_3_^2−^ and HCO_3_^−^. An iterative procedure was used to calculate the association constants. In the first iteration, it was assumed that the effective ionic strength, *I_E_*, was equal to the total ionic strength, *I_T_*, and the values for the (*n* − 1) association constants were calculated, where *n* is the number of differently protonated species of the studied acid. The procedure was repeated until two successive *I_E_* values did not differ by more than 0.001 units. The experimental values of the association constants of sodium with carbonate ion ($K_{1C}^{\prime }$) and acidic carbonate ions ($K_{2C}^{\prime }$) for NaCl-NaHCO_3_-H_2_O ternary systems will be used later for sodium speciation in the NaCl-NaHCO_3_-Na_2_HPO_4_-Na_2_SO_4_-H_2_O multicomponent electrolyte system. Speciation software packages available in the specialized literature were used for the calculations and graphic representations of the compositions of multicomponent mixtures [11,12,13,14,26].

### 2.1. Determination of Mean Activity Coefficients

Table 2 presents the experimental values of the mean activity coefficients of sodium chloride, $\gamma_{\mathrm{NaCl}}$, in the NaCl-NaHCO_3_-H_2_O ternary system for different compositions and constant total ionic strengths, and the experimental values of the mean activity coefficients of sodium chloride, $\gamma_{\mathrm{NaCl}}^{0}$, in the NaCl-H_2_O binary system. To facilitate the comparison of the obtained results with other data from the literature, concentrations and ionic strengths are expressed in mol/kg.

The $\gamma_{\mathrm{NaC}l}^{0}$ values obtained experimentally are comparable to the mean activity coefficients of sodium chloride values calculated with the Pitzer–Millero equation and using the SIT model [27], with a −0.9 to 0.1% relative standard deviation. The results show that the activity coefficients of sodium chloride in the ternary system are smaller than the mean experimental activity coefficient in pure sodium chloride,${\;\gamma }_{\mathrm{NaCl}}$ < $\gamma_{\mathrm{NaCl}}^{0}$, over the entire field of ionic states studied. This is due to the presence of sodium acidic carbonate in the system.

### 2.2. Determination of Harned’s Coefficient

The association model is applicable with good results when the ionic associations are significant. An assessment of the magnitude of the ionic associations is made based on the criterion of compliance with Harned’s rule. When this rule is respected, the extent of ionic associations is small [16].

Harned’s rule is given in Equation (1):

(1) $$\log\gamma_{\mathrm{NaCl}}=\log\gamma_{\mathrm{NaCl}}^{0}-\alpha_{12}I_{T}(1-X_{\mathrm{NaCl}})$$

where:

*I_T_*—total ionic strength;

$\gamma_{{\mathrm{NaCl}}^{-}}$—activity coefficient of sodium chloride in a mixture of electrolytes at *I_T_* ionic strength;

$\gamma_{{\mathrm{NaCl}}^{-}}^{0}$—mean activity coefficient of sodium chloride in the NaCl-H_2_O binary system, at *I_T_* ionic strength;

*α*_12_—Harned’s coefficient;

*X*_NaCl_—ionic strength fraction of sodium chloride ($X_{\mathrm{NaCl}}=m_{\mathrm{NaCl}}/I_{T}$).

**Figure 1:**
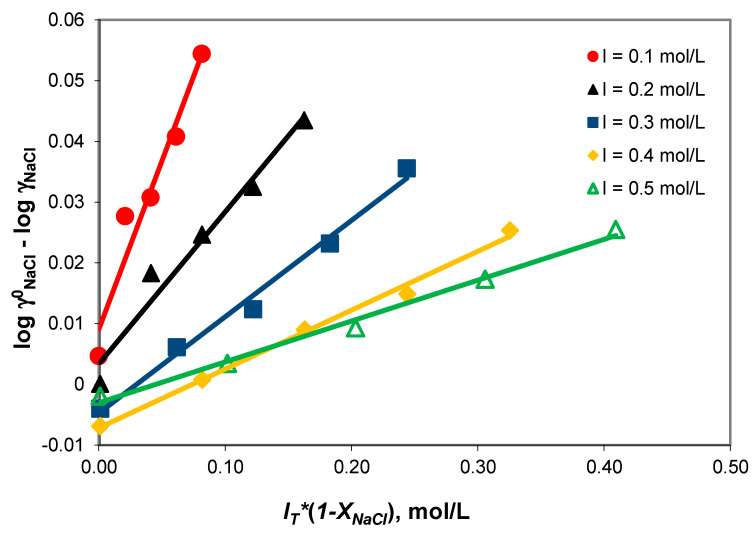
Compliance with Harned’s rule verification for ionic strengths in the 0.1–0.5 mol/kg range.

Figure 1 shows that the log$\gamma_{\mathrm{NaCl}}$ − log$\gamma_{\mathrm{NaCl}}^{0}$ term varies linearly with I_T_*(1 − *X*_NaCl_) at a 0.1–0.5 mol/kg constant total ionic strength. The NaCl-NaHCO_3_-H_2_O ternary system obeys Harned’s rule. The compliance of Harned’s rule in the NaCl-NaHCO_3_-H_2_O ternary system (Table 3) indicates weak associations of the sodium ion with the acidic carbonate ions.

The values of Harned’s coefficient (α_12_), obtained experimentally at a 0.5 mol/kg total ionic strength, are similar to those obtained by Butler and Huston [18]. There are no values of Harned’s coefficient available in the literature for total ionic strengths lower than 0.5 mol/kg.

**Figure 2:**
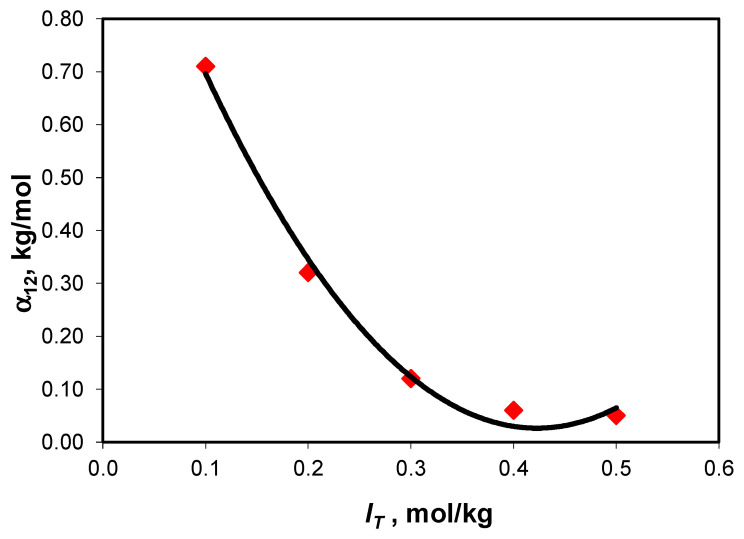
Variation in Harned’s coefficient with total ionic strength for the NaCl-NaHCO_3_-H_2_O ternary system at 25 °C.

Harned’s coefficient depends significantly on the total ionic strength at low ionic strength values, but it becomes almost invariable at ionic strength values larger than 0.4 mol/kg (Figure 2), a behavior also reported by Sirbu et al. [28] for the NaCl-Na_2_SO_4_-H_2_O ternary system.

**Figure 3:**
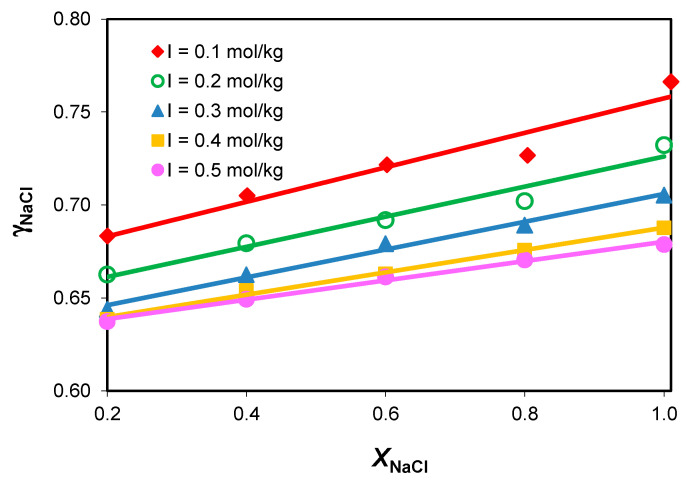
Variation in mean activity coefficients with composition in the NaCl-NaHCO_3_-H_2_O ternary system, at different constant total ionic strength values.

The influence of the medium composition on the sodium chloride activity coefficients, $\gamma_{\mathrm{NaCl}}$, in the NaCl-NaHCO_3_-H_2_O ternary system at constant total ionic strength and different compositions is presented in Figure 3.

The mean activity coefficient values in the NaCl-NaHCO_3_-H_2_O ternary system increase linearly with the increase in the sodium chloride concentration and the decrease in the sodium acidic carbonate level in the medium.

### 2.3. Determination of Association Constants

The association constant of sodium ions with carbonate ions ($K_{1C}^{\prime }$) and the association constant of sodium ions with acidic carbonate ions ($K_{2C}^{\prime }$) for the NaCl-NaHCO_3_-H_2_O ternary system were determined as functions of the effective ionic strength.

**Figure 4:**
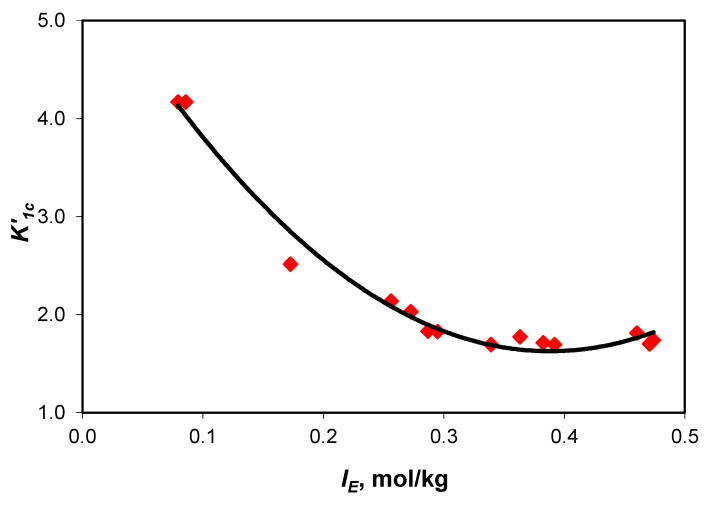
Variation in association constant of sodium ions with carbonate ions ($K_{1C}^{\prime }$) values with the effective ionic strength curve equation y = (26.32 ± 2.22) x^2^ − (20.43 ± 1.28) x + (5.59 ± 0.17) with R^2^ = 0.9804 and 0.132 standard error.

**Figure 5:**
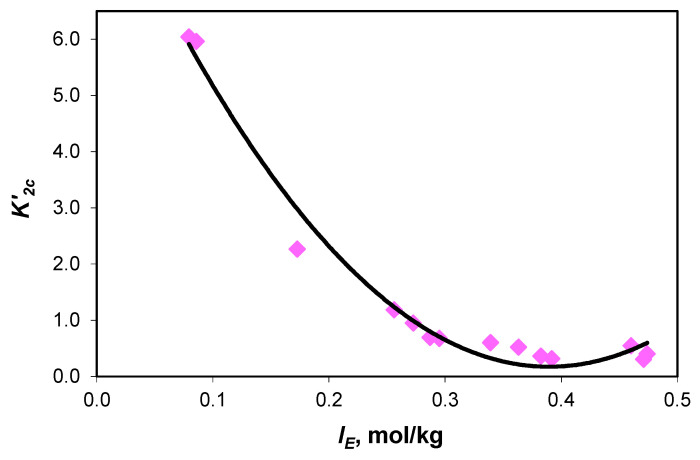
Variation in association constant of sodium ions with acidic carbonate ions ($K_{2C}^{\prime }$) values with the effective ionic strength curve equation y = (59.60 ± 4.98) x^2^ − (46.44 ± 2.86) x + (9.22 ± 0.37), with R^2^ = 0.9812 and 0.295 standard error.

The dependence of the association constants upon the effective ionic strength (Figure 4 and Figure 5) is described by Equations (2) and (3).

(2) $$K_{1C}^{\prime }=5.59-20.43\times I_{E}+26.32\times I_{E}^{2}\;\;\;R^{2}=0.9804$$

(3) $$K_{2C}^{\prime }=9.22-46.44\times I_{E}+59.60\times I_{E}^{2}\;\;\;R^{2}=0.9812$$

The experimental values of the association constant of sodium ions with carbonate ions ($K_{1C}^{\prime }$) and the association constant of sodium ions with acidic carbonate ions ($K_{2C}^{\prime }$) are presented in Table 4. Extrapolated to infinite dilution, the values of the association constants are $K_{1C}^{\prime }=14.41$ and $K_{2C}^{\prime }=1.71$.

**Figure 6:**
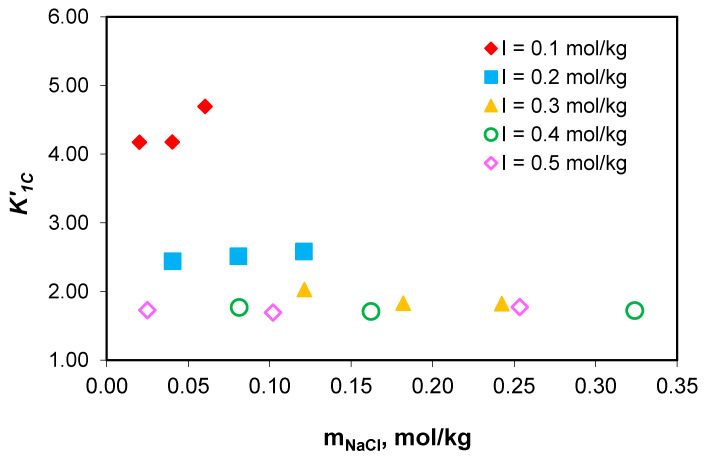
Variation in the association constant of sodium ions with carbonate ion ($K_{1C}^{\prime }$) values with sodium chloride concentration at 0.1–0.5 mol/kg total ionic strength range.

**Figure 7:**
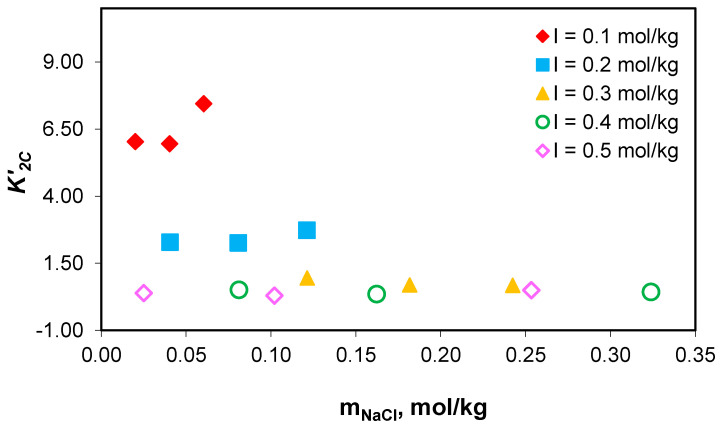
Variation in the association constant of sodium ions with acidic carbonate ions values with sodium chloride concentration at 0.1–0.5 mol/kg total ionic strength range.

The possibility of using the $K_{1C}^{\prime }$ and $K_{2C}^{\prime }$ association constant values measured in the NaCl-NaHCO_3_-H_2_O ternary system to determine the distribution of sodium among the chemical species present in the growth medium of *Chlorella homosphaera 424* algae was verified. This was carried out by demonstrating the association constants’ independence of the composition of the medium, at constant total ionic strength, as demonstrated in Figure 6 and Figure 7.

The studies carried out show that the values of the association constant of sodium ions with carbonate ions ($K_{1C}^{\prime }$) and the association constant of sodium ions with acidic carbonate ions ($K_{2C}^{\prime }$) do not depend on the composition of the medium, but only on the effective ionic strength. The obtained results prove that the association constants of sodium with carbonate ion ($K_{1C}^{\prime }$) and acidic carbonate ions ($K_{2C}^{\prime }$) for NaCl-NaHCO_3_-H_2_O ternary systems can be used for sodium speciation in the NaCl-NaHCO_3_-Na_2_HPO_4_-Na_2_SO_4_-H_2_O multicomponent electrolyte system, and similarly with algal media growth.

**Figure 8:**
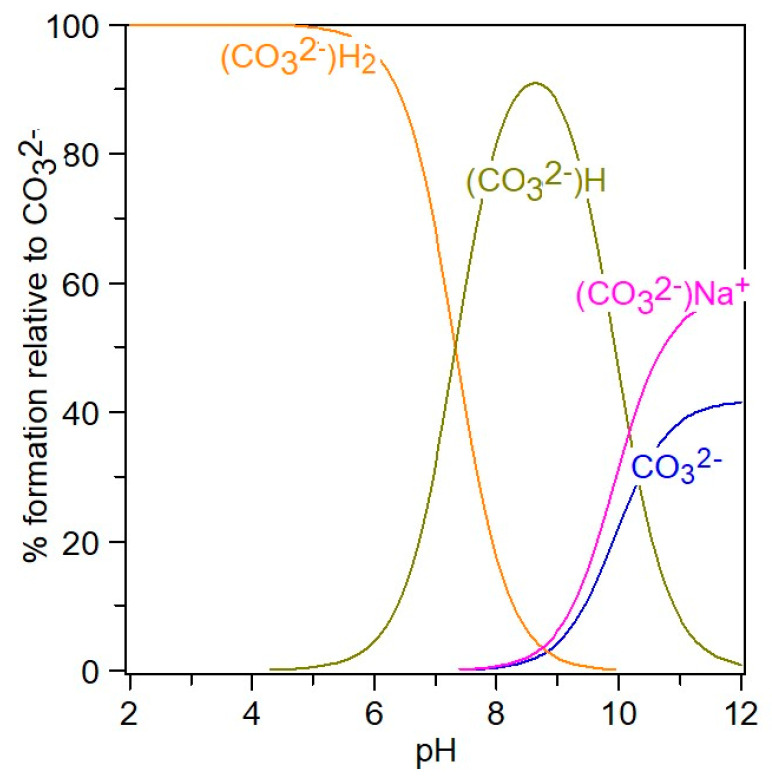
Distribution of CO_3_^2−^ species in 4–12 pH range, at *I_T_* = 0.5 mol/kg.

**Figure 9:**
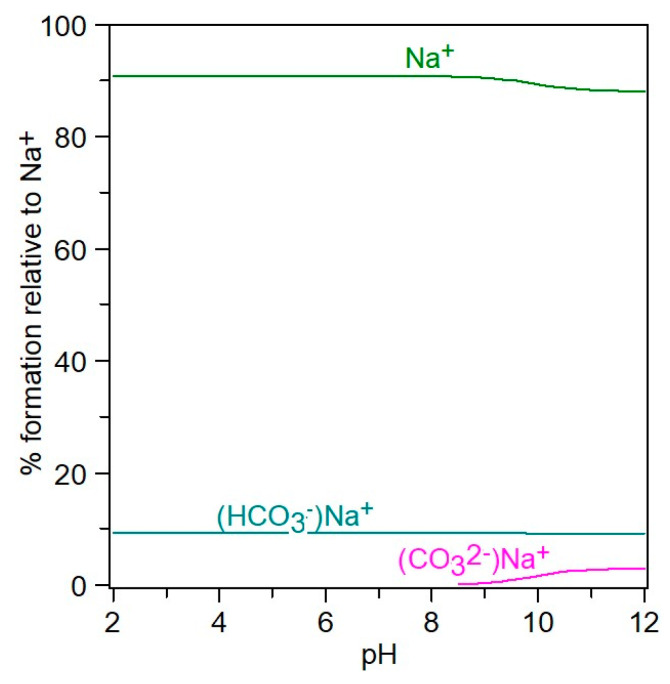
Distribution of Na^+−^ species in 4–12 pH range, at *I_T_* = 0.5 mol/kg.

Chemical speciation can be visualized with the aid of *Hyperquad simulation and speciation* (HySS2009) [11,12], a utility program for the investigation of equilibria involving soluble and partially soluble species, available in electronic format at http://www.hyperquad.co.uk/hyss.htm (accessed on 20 September 2023). HySS, a computer program compatible with the Windows operating system, allows both the simulation of titration curves and the obtaining of speciation diagrams. Input data for speciation are the reactants’ total concentrations and association constants. The distribution of free ions and ion pairs present in the NaCl-NaHCO_3_-H_2_O ternary system, at different *p*H values, is presented in Figure 8 and Figure 9.

In the 4–12 pH range (Figure 8), starting from H_2_CO_3_, the CO_3_^2−^ species are present in the form of HCO_3_^−^, NaCO_3_^−^, and free CO_3_^2−^, meaning that at pH = 12, the carbonate is associated with sodium in a proportion of 54%, while 44% CO_3_^2−^ is free. It can be seen from Figure 9 that in the NaCl-NaHCO_3_-H_2_O system, as the pH increases, the percentage of associated sodium in the form of NaCO_3_^−^ and NaHCO_3_ increases, reaching up to 12% at pH 12, and the unassociated sodium decreases to 88%. At pH 8, it is observed that there is 90% free sodium, and 10% associated sodium in the form of NaHCO_3_.

## 3. Materials and Methods

### 3.1. Reagents

Sodium chloride (≥99.8% purity, Sigma-Aldrich, Darmstadt, Germany) and sodium acidic carbonate ions (99.5–100.5% purity, Merck, Darmstadt, Germany) were used for experiments without further purification. All solutions were prepared with ultrapure water (18.2 MΩ), boiled, and cooled.

### 3.2. Equipment

A multifunctional DL50 Graphix (Mettler Toledo, Schwerzenbach, Switzerland) potentiometric system, equipped with a thermostated cell, DX-223 (Mettler Toledo) sodium ion-selective electrode, and EA 306- Cl (Metrohm, Herisau, Switzerland) chloride ion-selective electrode fitted with a DX200 (Mettler Toledo, Schwerzenbach, Switzerland) double-junction Ag/AgCl reference electrode were used in the experimental studies. An Ecoline RE 104 thermostatic recirculation bath (Lauda, Lauda-Königshofen, Germany) with a precision of ±0.1 °C ensured isothermal conditions; weighing was carried out on a XS 204/M (Mettler Toledo, Greifensee, Switzerland) analytical balance with a precision of ±0.0001 g; sodium chloride was dried in an oven (Memmert, Büchenbach, Germany) and cooled in desiccator over silica gel before use. Ultrapure water was obtained using EassyPure RoDi water purification equipment (Thermo Fisher Scientific, Waltham, MA, USA).

Class A glassware was used for the preparation of all solutions.

The solutions *p*H values were measured with a P901 CONSORT portable pH-meter (Turnhout, Belgium), equipped with DG111 (Mettler Toledo, Schwerzenbach, Switzerland) combined glass electrode. A 2-point calibration routine with 7.00 and 10.00 pH buffers was employed to adjust the slope and the asymmetry potential of the measuring device. The hydrogen ion activity scale (pH = −log a_H_) was used.

Density determinations were performed with a Gay-Lussac pycnometer (Roth). The pycnometer was calibrated with double-distilled water.

All determinations were carried out at 25 °C. The cell temperature was monitored with a calibrated thermometer with an expanded uncertainty of 0.5 °C for a coverage factor k = 2 and a probability P = 95%.

### 3.3. Method

#### 3.3.1. Determination of Mean Activity Coefficients of Sodium Chloride in NaCl-H_2_O and NaCl-NaHCO_3_-H_2_O Systems

The mean activity coefficients of sodium chloride, both in pure sodium chloride and in a mixture of electrolytes, were determined according to Bates and Dickson [29], through two consecutive measurements of the electromotive voltage in cells with a liquid junction consisting of the indicating electrode and the same reference electrode and immersed in the electrolytic medium.

For the correct measurement of the mean activity coefficients of sodium chloride, the sodium and chloride ion-selective electrodes were calibrated each time before use in sodium chloride standard solutions, using two electrochemical cells of the I and II type [29].

$$\mathrm{Cell}\;I:\;\mathrm{Ag},\;\mathrm{AgCl}|\;\mathrm{KCl}\;(3\;M)||\;{\mathrm{NH}}_{4}{\mathrm{NO}}_{3}\;(1\;M)|\;\mathrm{NaCl}\;(m_{\mathrm{NaCl}})|\;{\mathrm{Na}}^{+}-\mathrm{ISE}$$

$$\mathrm{Cell}\;\mathrm{II}:\;\mathrm{Ag},\;\mathrm{AgCl}|\;\mathrm{KCl}\;(3\;M)||\;{\mathrm{NH}}_{4}{\mathrm{NO}}_{3}\;(1\;M)|\;\mathrm{NaCl}\;(m_{\mathrm{NaCl}})|\;{\mathrm{Cl}}^{-}-\mathrm{ISE}$$

The electromotive force corresponding to each cell is described by Equations (4) and (5):

(4) $$E_{Na^{+}}=E_{Na^{+}}^{0}+S_{Na^{+}}{}^{*}\log{\;a}_{Na^{+}}-E_{ref}+E_{j}$$

(5) $$E_{Cl^{-}}=E_{Cl^{-}}^{0}-S_{Cl^{-}}{}^{*}\log\;a_{Cl^{-}}-E_{ref}+E_{j}$$

where:

$E_{Na^{+}}^{0}$, $E_{Cl^{-}}^{0}$—the standard potentials of cells I and II;

$S_{Na^{+}}$, $S_{Cl^{-}}$—the slopes of the sodium and chloride electrodes, respectively;

*E_j_*—the junction potential;

*E_ref_*—the potential of the reference electrode;

*a*_Na_^+^—sodium ion activity;

*a*_Cl_*^−^*—chloride ion activity.

The difference between the electromotive force of the I and II cells is equal to the electromotive force of cell III, without liquid junction:

$$\mathrm{Cell}\;\mathrm{III}:\;{\mathrm{Na}}^{+}-\mathrm{ISE}|\mathrm{NaCl}\;(m_{\mathrm{NaCl}})|{\mathrm{Cl}}^{-}-\mathrm{ISE}$$

From a stock solution of 1 mol/L sodium chloride, a series of fourteen standard solutions in the 0.0001–0.5 mol/L concentration range was prepared. The electromotive force of the two cells I and II were measured consecutively, practically under the same conditions: the same solution, the same reference electrode, the same temperature, with a time difference between determinations of maximum 5 min. The temperature of the solutions was kept constant at 25 °C, with a precision of ± 0.1 °C.

Calibration data were fitted to first-degree equations:

(6) $$E_{\mathrm{NaCl}}=S_{\mathrm{NaCl}}{*a}_{\mathrm{NaCl}}-E_{\mathrm{NaCl}}^{0}=-114.14*a_{\mathrm{NaCl}}-108.29,\;R^{2}=0.9992$$

(7) $$a_{\mathrm{NaCl}}=\log\left(m_{\mathrm{NaCl}}\times \gamma_{\mathrm{NaCl}}^{0}\right)$$

(8) $$S_{\mathrm{NaCl}}=S_{Na^{+}}+S_{Cl^{-}}$$

(9) $$E_{\mathrm{NaCl}}^{0}=E_{Cl^{-}}^{0}-E_{Na^{+}}^{0}$$

where:

$m_{{\mathrm{NaCl}}^{-}}$—sodium chloride molal concentration,

$\gamma_{{\mathrm{NaCl}}^{-}}^{0}$—mean activity coefficient of sodium chloride in pure sodium chloride,

$S_{Na^{+}}$, ${\;S}_{Cl^{-}}$—slopes of the sodium and chloride electrodes, respectively,

$E_{Na^{+}}^{0}$, $E_{Cl^{-}}^{0}$—standard potentials of cells I and II,

$E_{NaCl}^{0}$—standard potential of cell III.

At concentrations higher than 0.01 mol/L, the mean activity coefficients of sodium chloride were calculated with the extended Debye–Hückel Equation (10).

(10) $$\log\gamma_{\mathrm{NaCl}\;}^{0}=\log\gamma_{D-H}+C*I_{T}+D*{I_{T}^{2}}^{}=-\frac{0.509*{{I_{T}^{0.5}}_{}}^{}}{1+0.328*4I_{T}^{0.5}}+0.04*I_{T}+0.03*I_{T}^{2}$$

The $\log\gamma_{D-H}$ term is given by the Debye–Hückel relation (11)

(11) $$\log\gamma_{D-H\;}=-\frac{A\left|z_{+}z_{-}\right|I^{0.5}}{1+Ba{I^{0.5}}^{}}\;$$

where:

A, B—the Debye–Hückel constants whose values at 25 °C are 0.5091 and 0.3286, respectively [29];

$z_{+},z_{-}$—the cation and anion charges;

*I*—ionic strength of the solution;

*a*—dimensional parameter of the ion. For an approximate calculation, in the case of univalent ions, a = 4Ǻ [29].

The values of the constants *C* and *D* were obtained via the graphic representation of potential *E*′ = *f*(*I*) = *f*(*m*), where

(12) $$E^{\prime }=E_{\mathrm{NaCl}}^{0}-S_{\mathrm{NaCl}}*C*I-S_{\mathrm{NaCl}}*D*I^{2}$$

The experimental data are modeled by a polynomial second degree equation:

(13) $$E^{\prime }=1.24*I^{2}-8.04*I-108.6\;\;R^{2}=0.9604$$

The values of the constants *C* and *D* are calculated from the parameters of the polynomial regression curve as follows:

$$C=\frac{8.04}{S_{\mathrm{NaCl}}}=\frac{8.04}{114}=0.07$$

$$D=\frac{1.24}{S_{NaCl}}=\frac{1.24}{114}=0.01$$

Electrochemical cells of the type IV and V were used to determine the mean activity coefficients of sodium chloride ($\gamma_{\mathrm{NaCl}}$) in the NaCl-NaHCO_3_-H_2_O ternary system at 0.1–0.5 mol/kg total ionic strength.

$$\mathrm{Cell}\;\mathrm{IV}:\;\mathrm{Ag},\;\mathrm{AgCl}|\;\mathrm{KCl}\;(3\;M)||\;{\mathrm{NH}}_{4}{\mathrm{NO}}_{3}\;(1\;M)|\mathrm{NaCl}\;(m_{\mathrm{NaCl}}),\;{\mathrm{NaHCO}}_{3}\;(m_{{\mathrm{NaHCO}}_{3}})|{\mathrm{Na}}^{+}\text{-}\mathrm{ISE}$$

$$\mathrm{Cell}\;V:\;\mathrm{Ag},\;\mathrm{AgCl}|\;\mathrm{KCl}\;(3\;M)||\;{\mathrm{NH}}_{4}{\mathrm{NO}}_{3}\;(1\;M)|\;\mathrm{NaCl}\;(m{}_{\mathrm{NaCl}}),\;{\mathrm{NaHCO}}_{3}\;(m{}_{{\mathrm{NaHCO}}_{3}})|{\mathrm{Cl}}^{-}\text{-}\mathrm{ISE}$$

where:

*m*${}_{{\mathrm{NaHCO}}_{3}}$—the sodium acidic carbonate molal concentration.

The total ionic strength of the medium is described by the equation:

(14) $$I_{T}=m_{\mathrm{NaCl}}+m_{{\mathrm{NaHCO}}_{3}}$$

The mean activity coefficients of sodium chloride in the presence of sodium acidic carbonate ($\gamma_{\mathrm{NaCl}}$) were calculated with the equation:

(15) $$\log\gamma_{\mathrm{NaCl}}=\frac{E_{\mathrm{NaCl}}-E^{0}+S_{Na^{+}}*\log m_{Na^{+}}-S_{Cl^{-}}*\log m_{Cl^{-}}}{S_{\mathrm{NaCl}}}\;$$

#### 3.3.2. Determination of Association Constants for the NaCl-NaHCO_3_-H_2_O Ternary System

The association constants of sodium ions with carbonate ions ($K_{1C}^{\prime }$) and acid carbonate ions ($K_{2C}^{\prime }$), respectively, were determined in a series of NaCl-NaHCO_3_-H_2_O solutions of different compositions at 0.1–0.5 mol/kg total ionic in the 8.34–8.95 *p*H range (Table 1).

An iterative procedure was used to calculate the association constants [17,18,19]. In the first iteration, the effective ionic strength, *I_E_*, was assumed to be equal to the total ionic strength, *I_T_*, and the constant $K_{1C}^{\prime }$ was considered equal to 3.162 [18]. Iterations were developed using a computer and were repeated until two successive values of *I_E_* did not differ by more than 0.001 units [18].

Starting from the hypothesis that at constant total ionic strength, the modification of the mean activity coefficients occurs only as a result of ion pairing, it can be written that

(16) $$[Na^{+}{]}_{F}*\left[Cl^{-}\right]*\gamma_{\mathrm{Na}*}\gamma_{\mathrm{Cl}}=(m_{\mathrm{NaCl}}+m_{\mathrm{NaHC}O_{3}})m_{\mathrm{NaCl}*}\gamma_{\mathrm{NaCl}}^{2}$$

where $m_{\mathrm{NaCl}}+m_{\mathrm{NaHC}O_{3}}=[Na^{+}{]}_{T}$ represents the total concentration of sodium ions and $[Na^{+}{]}_{F}$ is the equilibrium concentration of free sodium ions. 

Since chloride ions do not form ion pairs, [Cl^−^] = m_NaCl_ and individual activity coefficients of sodium and chloride ions can be written as γNaCl0=γNaγCl12.

So, Equation (16) can be reformulated as

(17) $$[Na^{+}{]}_{F}{*\left(\gamma_{\mathrm{NaCl}}^{0}\right)}^{2}=[Na^{+}{]}_{T}*\gamma_{\mathrm{NaCl}}^{2}$$

Thus, $\gamma_{\mathrm{NaCl}}^{0}$ can be expressed as the mean activity coefficient of sodium chloride at an effective ionic strength, *I_E_* = *I_T_*. This ionic strength is expressed based on the equilibrium concentrations of the various ionic species:

(18) $$I_{E}=\frac{1}{2}\left(\left[Na^{+}{]}_{F}+[Cl^{-}]+[\mathrm{HC}O_{3}^{-}]+4[CO_{3}^{2-}]+[\mathrm{NaC}O_{3}^{-}\right]\right)$$

The equilibrium concentration values are obtained from the protonation and association equilibria:

(19) $$\left[\mathrm{HC}O_{3}^{-}\right]=K_{a_{2}C}\left[H_{3}O^{+}\right]*[CO_{3}^{2-}]$$

(20) $$\left[\mathrm{NaC}O_{3}^{-}]=K_{1C}^{\prime }[Na^{+}{]}_{F}*[CO_{3}^{2-}\right]$$

(21) $$\left[\mathrm{NaHC}O_{3}]=K_{2C}^{\prime }[Na^{+}{]}_{F}*[\mathrm{HC}O_{3}^{-}\right]$$

and from the mass balance equations for the three components:

(22) $$[Cl^{-}]=m_{\mathrm{NaCl}}$$

(23) $$[Na^{+}{]}_{F}+[\mathrm{NaHC}O_{3}]+[\mathrm{NaC}O_{3}^{-}]=[Na^{+}{]}_{T}$$

(24) $$[\mathrm{HC}O_{3}^{-}]+[CO_{3}^{2-}]+[\mathrm{NaC}O_{3}^{-}]+[\mathrm{NaHC}O_{3}]=[\mathrm{NaHC}O_{3}{]}_{T}$$

where:

$K_{a_{2}C}$—the second dissociation constant of H_2_CO_3_;

$K_{1C}^{\prime }$—the association constant of sodium ions with carbonate ions;

$K_{2C}^{\prime }$—the association constant of sodium ions with acidic carbonate ions.

The concentration of free sodium ions is calculated using Equation (25):

(25) $$[Na^{+}{]}_{F}=\frac{[Na^{+}{]}_{T}*{\left(\gamma_{\mathrm{NaCl}}\right)}^{2}}{{\left(\gamma_{\mathrm{NaCl}}^{0}\right)}^{2}}$$

After completing the iterative procedure, $K_{1C}^{\prime }$ was recalculated with the formula:

(26) $$K_{1C}^{\prime }=\frac{\left[\mathrm{NaC}O_{3}^{-}\right]}{\left[Na^{+}{]}_{F}*[CO_{3}^{2-}\right]}$$

## 4. Conclusions

The association constants of sodium ions with carbonate ions,${\;K}_{1C}^{\prime }$, and with acidic carbonate ions,${\;K}_{2C}^{\prime }$, respectively, in the NaCl-NaHCO_3_-H_2_O ternary system were determined experimentally as functions of the effective ionic strength at 0.1–0.5 mol/kg total ionic strengths, in the 8.34–8.95 *p*H range.

The studies carried out show that the values of the association constants do not depend on the composition of the medium, but only on the effective ionic strength. In conclusion, these values can be further used in NaCl-NaHCO_3_-Na_2_HPO_4_-Na_2_SO_4_-H_2_O multicomponent systems, finally giving access to the real-time distribution of sodium among the chemical species present in the growth medium of *Chlorella homosphaera 424* algae.

In the 0.1–0.5 mol/kg ionic strength range, the mean activity coefficient of NaCl in the ternary system, $\gamma_{\mathrm{NaCl}},$ is smaller than $\gamma_{\mathrm{NaCl}}^{0}$ in the binary system over the entire field of ionic strengths studied. This is due to the presence of NaHCO_3_ in the system, which confirms the hypothesis underlying the ionic association model, in the modified version proposed by Pytkowicz and Kester [16].

The NaCl-NaHCO_3_-H_2_O ternary system obeys Harned’s rule, which indicates weak associations of the sodium ion with the acidic carbonate ion.

At low ionic strength, Harned’s coefficient, α_12_, depends significantly on the total ionic strength, but it becomes almost invariable at ionic strengths larger than 0.4 mol/kg.

## Figures and Tables

**Table 1:** Existing data on the association constants of sodium with carbonate $\left(log\;K_{1C}^{\prime }\right)$ and acid carbonate ions $\left({log\;K}_{2C}^{\prime }\right)$, in different media, at 25 °C, available in the literature.

Technique	Medium	Ionic Strength (M)	$logK_{1C}^{\prime }$	${logK}_{2C}^{\prime }$	Reference
Na^+^ ISE Sodium amalgam electrode	NaCl	0.0	0.96	−0.30	[18]
		0.5	0.14	−0.41	
		1.0	0.27	−0.67	
		3.0	0.37	-	
*p*H electrode	Sea water	0.72	0.63	−0.55	[20]
Na^+^ ISE	Me_4_NCl	1.0	0.3971	-	[21]
		~0.11	0.26	-	[22]
		~0.18	0.31		
		0.19	0.23		
Na^+^ ISE	CsCl	0.0	1.29	-	[21]
		0.5	0.5024	-	
		1.0	0.3169	-	
		2.0	0.1586	-	
		3.0	0.1041	-	
		4.0	0.1124	-	
		5.0	0.1238	-	
		7.0	0.1566	-	
Na^+^ ISE	NaCl-[(CH_3_)_4_N]_2_BPDS-Me_4_NCl, for $K_{1C}^{\prime }$ NaCl-Me_4_NCl, for $K_{2C}^{\prime }$	0.0	1.28	0.53	[23]
		~0.24	0.55	0.10	
		0.34	-	0.03	
		0.49	0.40	-	
		~0.69	0.37	−0.11	
Na^+^ ISE	KCl-Me_4_NCl	0.0	0.38	-	[22]
		~0.09	0.30	-	
		~0.10	0.59	-	
		0.19	0.29	-	
		0.20	0.34	-	
		0.38	0.30	-	
		0.76	0.22	-	

**Table 2:** The experimental values of mean activity coefficients of sodium chloride in the ternary system, $\gamma_{\mathrm{NaCl}}$, and NaCl-H_2_O system,${\;\gamma }_{\mathrm{NaCl}}^{0}$, against the ionic strength fraction of sodium chloride (X_NaCl_), at constant total ionic strength, *I_T_*, and 25 °C.

*I_T_* (mol/kg)	X_NaCl_	m_NaHCO_3__ (mol/kg)	m_NaCl_ (mol/kg)	E_NaCl_ (mV)	$\gamma_{\mathrm{NaCl}}$	$\gamma_{\mathrm{NaCl}}^{0}$
0.10	0.2	0.08	0.02	60.74	0.6832	0.7662 (0.7659) *
	0.4	0.06	0.04	42.25	0.7050	
	0.6	0.04	0.06	31.21	0.7215	
	0.8	0.02	0.08	23.84	0.7266	
	1.0	0.00	0.10	15.57	-	
0.20	0.2	0.16	0.04	27.60	0.6626	0.7322 (0.7255) *
	0.4	0.12	0.08	9.43	0.6795	
	0.6	0.08	0.12	−1.36	0.6919	
	0.8	0.04	0.16	−9.11	0.7021	
	1.0	0.00	0.20	−16.73	-	
0.30	0.2	0.24	0.06	8.68	0.6438	0.7053 (0.7031) *
	0.4	0.18	0.12	−9.63	0.6625	
	0.6	0.12	0.18	−20.74	0.6792	
	0.8	0.06	0.24	−28.49	0.6891	
	1.0	0.00	0.31	−35.41	-	
0.40	0.2	0.32	0.08	−5.36	0.6385	0.6877 (0.6885) *
	0.4	0.24	0.16	−23.46	0.6541	
	0.6	0.16	0.24	−33.99	0.6629	
	0.8	0.08	0.32	−41.94	0.6757	
	1.0	0.00	0.41	−48.41	-	
0.51	0.2	0.41	0.10	−16.02	0.6372	0.6787 (0.6783) *
	0.4	0.30	0.20	−34.32	0.6494	
	0.6	0.20	0.30	−45.30	0.6614	
	0.8	0.10	0.40	−53.06	0.6704	
	1.0	0.00	0.51	−59.58	-	

* The values in brackets were calculated with Pitzer–Millero equation using SIT model [27].

**Table 3:** Values of Harned’s coefficient in the NaCl-NaHCO_3_-H_2_O ternary system, at different total ionic strengths, and 25 °C.

*I_T_* (mol/kg)	α_12_ (kg/mol)	α_12_ * (kg/mol)
0.1	0.71 ± 0.05	-
0.2	0.32 ± 0.09	-
0.3	0.12 ± 0.02	-
0.4	0.06 ± 0.01	-
0.5	0.049 ± 0.008	0.050 ± 0.009 *

* Taken from [18].

**Table 4:** Experimental values of association constant of sodium ions with carbonate ions ($K_{1C}^{\prime }$) and the association constant of sodium ions with acidic carbonate ions ($K_{2C}^{\prime }$).

*I_T_* (mol/kg)	*I_E_* (mol/kg)	m_NaCl_ (mol/kg)	m_NaHCO_3__ (mol/kg)	[Na^+^]_F_ (mol/kg)	[CO_3_^2−^] (mol/kg)	[HCO_3_^−^] (mol/kg)	[NaCO_3_^−^] (mol/kg)	*p*H	$K_{1C}^{\prime }$	$K_{2C}^{\prime }$
0.1	0.079	0.020	0.081	0.076	0.002	0.053	0.001	8.73	4.18	6.04
	0.086	0.040	0.060	0.081	0.003	0.038	0.001	8.95	4.18	5.96
	0.087	0.060	0.040	0.085	0.001	0.024	0.000	8.72	4.70	7.44
0.2	0.165	0.040	0.161	0.158	0.004	0.114	0.002	8.61	2.45	2.30
	0.173	0.081	0.121	0.168	0.003	0.085	0.001	8.54	2.52	2.27
	0.178	0.121	0.081	0.176	0.002	0.053	0.001	8.55	2.59	2.74
0.3	0.272	0.121	0.182	0.266	0.004	0.141	0.002	8.47	2.03	0.95
	0.287	0.182	0.121	0.283	0.002	0.098	0.001	8.43	1.83	0.69
	0.295	0.242	0.061	0.293	0.001	0.049	0.001	8.42	1.83	0.68
0.4	0.364	0.081	0.324	0.353	0.006	0.266	0.004	8.34	1.77	0.52
	0.383	0.162	0.243	0.374	0.005	0.207	0.003	8.35	1.71	0.36
	0.395	0.324	0.081	0.392	0.002	0.067	0.001	8.37	1.73	0.44
0.5	0.471	0.253	0.253	0.456	0.008	0.195	0.006	8.60	1.77	0.50
	0.475	0.102	0.408	0.456	0.010	0.344	0.008	8.45	1.69	0.30
	0.478	0.025	0.508	0.449	0.016	0.408	0.012	8.57	1.73	0.39

## Data Availability

Not applicable.
